# Selected patients can benefit more from the management of etoposide and platinum-based chemotherapy and thoracic irradiation-a retrospective analysis of 707 small cell lung cancer patients

**DOI:** 10.18632/oncotarget.14395

**Published:** 2016-12-31

**Authors:** Shoubo Cao, Shi Jin, Jing Shen, Jingyan Cao, Hua Zhang, Qingwei Meng, Chunyan Wang, Aiqi Zhang, Pei Zhang, Yan Yu

**Affiliations:** ^1^ Department of Medical Oncology, Harbin Medical University Cancer Hospital, Harbin, China

**Keywords:** small cell lung cancer, prognostic factors, subgroup analysis, inflammation, survival

## Abstract

The management of small cell lung cancer (SCLC) has reached a plateau. Etoposide and platinum-based chemotherapy plus thoracic irradiation remain the standard treatment strategy for SCLC. Our study aims to assess the potential prognostic factors of patients treated with etoposide and platinum-based chemotherapy and explore which group of patients can benefit more from standard treatment strategies. On univariate analysis, age>65 years, male patients, KPS (Karnofsky Performance Status)≤80 points, positive smoking history, anemia, lymphocyte counts≤1.65×10^9^/L, neutrophil to lymphocyte ratio (NLR)>3.18, lymphocyte to monocyte ratio (LMR)≤2.615, lactate dehydrogenase (LDH)>216.5 U/L, alkaline phosphatase (ALP)>119.5 U/L, absence of surgery, absence of thoracic irradiation, chemotherapy cycles<4, metastatic sites≥2 and extensive disease were correlated with a poor prognosis. Gender, KPS, chemotherapy cycles, thoracic irradiation, metastatic sites, LDH and tumor stage held statistical significance on multivariate analysis (*p*<0.05). High LDH was closely correlated with extensive disease, metastatic sites≥2, anemia, low LMR, high NLR and ALP levels. Subgroup analysis showed patients with male gender, KPS≤80 points, LDH≤216.5U/L, extensive disease and metastatic sites<2 could benefit more from ≥4 chemotherapy cycles. Patients with male gender, KPS>80 points, LDH≤216.5U/L, limited disease and metastatic sites<2 could benefit more from thoracic irradiation (*p*<0.05 on uni- and multivariate analysis). In conclusion, female patients, KPS>80 points, chemotherapy cycles≥4, thoracic irradiation, metastatic sites<2, LDH≤216.5U/L and limited disease were independent positive prognostic factors for SCLC patients treated with etoposide and platinum-based chemotherapy. Selected patients can benefit more from the management of ≥4 cycles of chemotherapy and thoracic irradiation.

## INTRODUCTION

SCLC is an aggressive disease with a high mortality and negative prognosis. Median survival is less than 1 year, with a 5-year survival of approximately 5% [1]. The management of SCLC has also reached a plateau for nearly 30 years. Etoposide and platinum-based chemotherapy plus thoracic irradiation remain the standard treatment strategy for SCLC [2]. However, treatment outcome is significantly different. Although patients received the same treatment strategies, many of them progressed quickly and even died soon. Systematic chemotherapy and thoracic radiotherapy have a limited role in these patients. On one hand, it leads to the increasing of economic burdens. On the other hand, it also adds some toxic reactions resulted from the treatment. The identification of patients who can benefit more from the treatment of chemotherapy and thoracic radiotherapy is of great value in clinics.

A number of retrospective studies have been performed to assess the prognostic roles of parameters in SCLC patients, but the patients enrolled are not treated with the same chemotherapy strategy and the results are not identical. Poor performance status, elevated serum LDH and extensive disease are usually associated with a short term survival in most studies [1, 3–5]. Alkaline phosphatase (ALP) [1, 5–8], age [9], gender [3, 8, 9], neutrophil [4, 9] and serum albumin [4, 6] were also found to be correlated with SCLC prognosis in some studies. The potential prognostic values of these parameters in SCLC patients treated with the same chemotherapy strategy remains unclear.

In clinics, we find the diagnosis of SCLC is usually accompanied by inflammation in most patients. Inflammation and the subsequent tumor development or tumor-elicited inflammation are critical steps for the development of cancers [10, 11]. Inflammation is often regarded as a feature of body innate immunity [11]. All types of immune cells can exist in the core, invasive margin of tumors as well as the adjacent tertiary lymphoid structures, and they can directly or indirectly affect tumor development through the secretion of cytokines [12–14]. Neutrophil to lymphocyte ratio (NLR), platelet to lymphocyte ratio (PLR) and lymphocyte to monocyte ratio (LMR), as key inflammatory markers, have been found to be associated with the prognosis of various malignancies including SCLC [15–21]. However, recent studies with small number of SCLC patients enrolled seldom assess the prognostic roles of some biochemical markers, lymphocyte, monocyte or LMR [16, 21]. And previous studies rarely involve the roles of lymphocyte, monocyte, NLR, PLR and LMR [1, 3–9]. We will take more serum biochemical markers into consideration to explore their prognostic values.

Our present study aims to assess the potential prognostic values of parameters in the presence of more serum biochemical markers in the whole group and variable subgroups of SCLC patients treated with the same chemotherapy strategy. But more importantly, we want to explore which group of patients can benefit more from the treatment of more cycles of etoposide and platinum-based chemotherapy and thoracic irradiation, and this is significant in clinics. It can help gain greater clinical benefits and survival time in the condition of smaller economic burdens and slighter toxic reactions.

## RESULTS

### Patient characteristics

**Figure 1:**
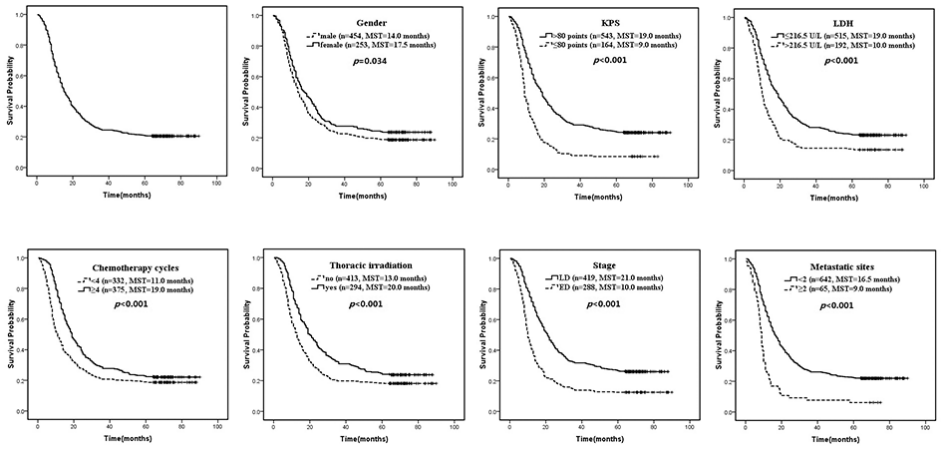
Overall survival and independent prognostic factors for 707 patients treated with etoposide and platinum-based chemotherapy MST, median survival time.

This study included 707 patients (454 men, 253 women). The mean age was 56.24±10.15 years (range 23-75). Smoking history was present in 62.5% (442/707) patients (Table 1). The most common metastatic sites at diagnosis were bone (58/707, 8.2%), liver (47/707, 6.6%), brain (44/707, 6.2%) and adrenal glands (21/707, 3.0%). The most common symptoms at presentation were cough (597/707, 84.4%), shortness of breath (244/707, 34.5%) and chest pain (243/707, 34.4%). Of the 707 patients, 288 (40.7%) patients suffered ED, 419 (59.3%) patients suffered LD. All patients were treated with etoposide and platinum-based chemotherapy, and only 294 (41.6%) patients received thoracic radiotherapy. The KPS was ≤80 points in 164 cases (23.2%) and >80 points in 543 cases (76.8%). The median time from initial symptoms presented to definitive diagnosis was 1.0 months. The median survival time (MST) was 15.0 months (95% CI, 13.552-16.448) for all cases and 145 patients were still alive at present (Figure 1). Of all 145 cases, 109 had LD and 36 had ED. The survival rates at 1-, 2- and 5-year were 59.0%, 34.1% and 20.8%, respectively. The median survival time and 1-, 2-, 5-year survival rate for LD and ED were 21.0 months, 71.6%, 43.7%, 26.5% and 10.0 months, 40.6%, 20.1%, 12.5%, respectively.

**Table 1:** Clinical characteristics of patients with SCLC

Features	n	%
Age (years)		
≤65	561	79.3
>65	146	20.7
Gender		
Male	454	64.2
Female	253	35.8
Symptoms initially presented		
Cough	597	84.4
Shortness of breath	244	34.5
Chest pain	243	34.4
Hemoptysis or blood in phlegm	233	33.0
Hoarseness	30	4.2
KPS (Karnofsky Performance Status)		
>80 point	543	76.8
≤80 points	164	23.2
Smoking history		
Yes	442	62.5
No	265	37.5
Family history of cancer		
Yes	116	16.4
No	591	83.6
Thoracic irradiation		
Yes	294	41.6
No	413	58.4
Surgery		
Yes	25	3.5
No	682	96.5
Chemotherapy cycles		
<4	332	47.0
≥4	375	53.0
Metastatic sites		
<2	642	90.8
≥2	65	9.2
Stage		
LD	419	59.3
ED	288	40.7

### The cutoff values for neutrophil, lymphocyte, monocyte, NLR, PLR, LMR, LDH and ALP

The mean (±SD) neutrophil, lymphocyte and monocyte counts, NLR, PLR, LMR, LDH and ALP were 4.55 (±2.01), 1.68 (±0.62), 0.49 (±0.23)×10^9^/L, 3.16 (±2.44), 174.90 (±107.97), 5.72 (±15.43), 210.75 (±136.20) U/L and 104.47 (±52.89) U/L, respectively. The ROC analysis showed the optimal LDH cut-off was 216.5 U/L (*p*<0.001, AUC, 0.611; 95% CI, 0.569–0.652). The patients were then divided into high (>216.5 U/L) and low LDH (≤216.5 U/L) groups. There were 192 (27.2%) patients in the high group and 515 (72.8%) in the low LDH group. The ALP of 119.5 U/L (*p*=0.231, AUC, 0.526; 95% CI, 0.483–0.569), neutrophil of 4.95×10^9^/L (*p*=0.577, AUC, 0.512; 95% CI, 0.469–0.555), lymphocyte of 1.65×10^9^/L (*p*=0.009, AUC, 0.557; 95% CI, 0.514–0.599), monocyte of 0.495×10^9^/L (*p*=0.183, AUC, 0.529; 95% CI, 0.486–0.571), NLR of 3.18 (*p*=0.037, AUC, 0.545; 95% CI, 0.503–0.588), PLR of 176.5 (*p*=0.194, AUC, 0.528; 95% CI, 0.486–0.571) and LMR of 2.615 (*p*=0.010, AUC, 0.556; 95% CI, 0.513–0.598) were selected as the optimal cut-off values.

### Serum biochemical examinations

Routine blood test and liver function evaluation were performed on each patient. The most common abnormalities were high levels of LMR (512/707,72.4%), lymphocyte (334/707, 47.2%), monocyte (321/707, 45.4%), ALT (268/707, 37.9%), neutrophil (248/707, 35.1%), LDH (192/707, 27.2%), platelet (160/707, 22.6%), low albumin level (105/707, 14.9%) and anemia (64/707, 9.1%). Carcinoembryonic antigen (CEA) and neuron specific enolase (NSE) were tested in 402 and 344 patients, and 29.1% (117/402) and 86.9% (299/344) patients showed an elevated level of CEA (>5 ng/ml) and NSE (>15.2 ng/ml), respectively.

### Univariate analysis

Each parameter was assessed by univariate analysis. Significant prognostic factors were age (*p*=0.025), gender (*p*=0.034), KPS (*p*<0.001), smoking history (*p*=0.009), anemia (*p*=0.026), lymphocyte counts (*p*=0.028), NLR (*p*=0.002), LMR (*p*=0.008), LDH (*p*<0.001), ALP (*p*=0.015), surgery (*p*=0.011), thoracic irradiation (*p*<0.001), chemotherapy cycles (*p*<0.001), metastatic sites (*p*<0.001) and tumor stage (*p*<0.001). We also assessed the prognostic values of CEA (n=402) and NSE (n=344). High NSE level was a negative prognostic factor on univariate analysis (>15.2 vs ≤15.2 ng/ml, 14.0 vs 24.0 months, *p*=0.009). No significant difference was found on variable CEA levels (>5 vs ≤5 ng/ml, 13.0 vs 15.5 months, *p*=0.219). These data showed the following factors were correlated with a poor prognosis: age>65 years, male patients, KPS≤80 points, positive smoking history, anemia (a hemoglobin value<12.0 g/dl in men and 11.0 g/dl in women), lymphocyte counts<1.65×10^9^/L, NLR>3.18, LMR≤2.615, LDH>216.5 U/L, ALP>119.5 U/L, NSE>15.2 ng/ml, absence of surgery, absence of thoracic irradiation, chemotherapy cycles**<**4, metastatic sites≥2 and extensive disease (Table 2). The variables examined in the final multivariate analysis were age, gender, KPS, smoking history, anemia, lymphocyte counts, NLR, LMR, LDH, ALP, surgery, thoracic irradiation, chemotherapy cycles, metastatic sites and tumor stage.

**Table 2:** Univariate analysis results of potential prognostic factors of SCLC patients treated with etoposide and platinum-based chemotherapy

Variables	O/N*	%	median survival time (months)	95% CI	p value
Age (years)					**0.025**
≤65	436/561	77.7	16.0	14.129-17.871	
>65	126/146	86.3	15.0	12.787-17.213	
Gender					**0.034**
Male	369/454	81.3	14.0	12.394-15.606	
Female	193/253	76.3	17.5	14.090-20.910	
KPS					**<0.001**
>80 point	412/543	75.9	19.0	16.943-21.057	
≤80 points	150/164	91.5	9.0	8.105-9.895	
Smoking history					**0.009**
Yes	364/442	82.4	14.0	12.640-15.360	
No	198/265	74.7	18.5	15.367-21.633	
Family history of cancer					0.180
Yes	98/116	84.5	13.0	9.047-16.953	
No	464/591	78.5	15.5	14.011-16.989	
Anemia					**0.026**
Yes	55/64	85.9	11.5	8.070-14.930	
No	507/643	78.8	16.0	14.469-17.531	
RBC					0.184
normal	500/629	79.5	16.0	14.485-17.515	
Below normal	62/78	79.5	10.0	6.634-13.366	
WBC (×109/L)					0.135
≤10	522/652	80.1	15.0	13.344-16.656	
>10	40/55	72.7	19.0	9.929-28.071	
Platelet					0.662
≤300	438/547	80.1	15.0	13.472-16.528	
>300	124/160	77.5	14.5	11.143-17.857	
Neutrophil (×109/L)					0.518
≤4.95	364/495	79.3	16.0	14.125-17.875	
>4.95	198/248	79.8	14.0	12.404-15.596	
Lymphocyte (×109/L)					**0.028**
≤1.65	303/373	81.2	13.0	11.675-14.325	
>1.65	259/334	77.5	18.0	15.442-20.558	
Monocyte (×109/L)					0.117
≤0.495	303/386	78.5	17.5	15.531-19.469	
>0.495	259/321	80.7	14.0	12.465-15.535	
NLR					**0.002**
≤3.18	357/462	77.3	17.0	14.894-19.106	
>3.18	205/245	83.7	13.0	11.014-14.986	
PLR					0.092
≤176.5	350/445	78.7	17.0	15.211-18.789	
>176.5	212/262	80.9	13.0	11.601-14.399	
LMR					**0.008**
≤2.615	161/195	82.6	12.0	10.422-13.578	
>2.615	401/512	78.3	17.0	15.152-18.848	
AST (U/L)					0.194
≤40	495/623	79.5	16.0	14.589-17.411	
>40	67/84	79.8	9.5	7.404-11.596	
ALT (U/L)					0.574
≤40	351/439	80.0	16.5	14.492-18.508	
>40	211/268	78.7	14.0	12.102-15.898	
LDH (U/L)					**<0.001**
≤216.5	396/515	76.9	19.0	16.737-21.263	
>216.5	166/192	86.4	10.0	8.869-11.131	
ALP (U/L)					**0.015**
≤119.5	431/547	78.8	16.5	14.737-18.263	
>119.5	131/160	81.9	12.0	9.717-14.283	
Serum albumin (g/L)					0.378
≤40	85/105	81.0	14.0	9.816-18.184	
>40	477/602	79.2	15.5	14.018-16.982	
Globulin (G, g/L)					0.123
≤35	522/651	80.2	15.0	13.626-16.374	
>35	40/56	71.4	20.0	12.055-27.945	
Surgery					**0.011**
Yes	15/25	60.0	31.0	0.000-69.353	
No	547/682	80.2	15.0	13.707-16.293	
Thoracic irradiation					**<0.001**
Yes	224/294	76.2	20.0	16.849-23.151	
No	338/413	81.8	13.0	11.662-14.338	
Chemotherapy cycles					**<0.001**
<4	270/332	81.3	11.0	9.215-12.785	
≤4	292/375	77.9	19.0	16.919-21.081	
Metastatic sites					**<0.001**
<2	501/642	78.0	16.5	14.969-18.031	
≤2	61/65	93.8	9.0	8.022-9.978	
Stage					**<0.001**
LD	310/419	74.0	21.0	18.668-23.332	
ED	252/288	87.5	10.0	9.050-10.950	

* O/N=observed death number/total patient number in each group

### Multivariate analysis

The multivariate analysis showed gender (*p*=0.027), KPS (*p*<0.001), LDH (*p*=0.001), chemotherapy cycles (*p*=0.005), thoracic irradiation (*p*=0.013), metastatic sites (*p*=0.015) and tumor stage (*p*<0.001) were statistically significant (Table 3). The hazard ratios of death were observed for LDH≥216.5U/L (1.415 fold increase compared to LDH<216.5 U/L), KPS≤80 points (1.834 fold increase compared to KPS>80 points), chemotherapy cycles<4 (1.276 fold increase compared to chemotherapy cycles≥4), absence of thoracic irradiation (1.25 fold compared to the presence of thoracic irradiation), extensive disease (1.428 fold compared to limited disease). Female patients, KPS>80 points, LDH≤216.5U/L, chemotherapy cycles≥4, thoracic irradiation, metastatic sites**<**2 and limited disease were independent positive prognostic factors for long-term survival of SCLC patients treated with etoposide and platinum-based chemotherapy.

**Table 3:** Multivariate analysis of risk factors for SCLC patients treated with etoposide and platinum-based chemotherapy

Variables	Hazard ratio	95% CI	*P* value
Gender	0.809	0.670-0.976	**0.027**
Age	0.985	0.802-1.210	0.888
KPS	1.834	1.498-2.245	**<0.001**
Smoking history	1.148	0.954-1.382	0.145
Anemia	1.060	0.794-1.416	0.691
Lymphocyte	0.879	0.729-1.059	0.176
NLR	1.030	0.837-1.267	0.780
LMR	1.053	0.848-1.307	0.641
LDH	1.415	1.161-1.724	**0.001**
ALP	1.002	0.818-1.228	0.984
Surgery	0.725	0.429-1.226	0.231
Chemotherapy cycles	0.784	0.660-0.931	**0.005**
Thoracic irradiation	0.800	0.671-0.954	**0.013**
Metastatic sites	1.440	1.073-1.934	**0.015**
Stage	1.428	1.177-1.732	**<0.001**

### The association between LDH and clinicopathological characteristics

LDH was identified to be serum independent prognostic factor of the whole SCLC patients on uni- and multivariate analysis. The optimal cutoff points to assess the difference of overall survival were 216.5 U/L for LDH. Using the optimal cutoff points, we divided all patients into two groups to evaluate the association of LDH levels and clinicopathological characteristics of SCLC patients (Table 4). We found that high levels of LDH was correlated with the presence of anemia (yes vs no, 39.1% vs 26.0%, *p*=0.025) and high NLR (yes vs no, 35.1% vs 22.9%, *p*=0.001), low LMR (yes vs no, 42.1% vs 21.5%, *p*<0.001), high ALP level (yes vs no, 36.2% vs 24.5%, *p*<0.001), extensive disease (yes vs no, 42.0% vs 16.9%, *p*<0.001) and more metastatic sites at diagnosis (yes vs no, 55.4% vs 24.3%, *p*<0.001). High LDH could be more frequently seen in patients with extensive disease, metastatic sites≥2, anemia, low LMR and high NLR levels.

**Table 4:** Correlation of the variable LDH level with the clinicopathological characteristics of SCLC patients

Variable	LDH (U/L)		
	≤216.5n (%)	>216.5n (%)	*p* value
Age (years)			0.051
≤65	418(74.5)	143(25.5)	
>65	97(66.4)	49(33.6)	
Gender			0.449
Male	335(73.8)	119(26.2)	
Female	180(71.1)	73(28.9)	
KPS			0.274
>80 points	401(73.8)	142(26.2)	
≤80 points	114(69.5)	50(30.5)	
Smoking history			0.224
Yes	315(71.3)	127(28.7)	
No	200(75.5)	65(24.5)	
Anemia			**0.025**
Yes	39(60.9)	25(39.1)	
No	476(74.0)	167(26.0)	
Lymphocyte(×109/L)			0.426
≤1.65	267(71.6)	106(28.4)	
>1.65	248(74.3)	86(25.7)	
NLR			**0.001**
≤3.18	356(77.1)	106(22.9)	
>3.18	159(64.9)	86(35.1)	
LMR			**<0.001**
≤2.615	113(57.9)	82(42.1)	
>2.615	402(78.5)	110(21.5)	
ALP (U/L)			**0.003**
≤119.5	413(75.5)	134(24.5)	
>119.5	102(63.8)	58(36.2)	
Stage			**<0.001**
LD	348(83.1)	71(16.9)	
ED	167(58.0)	121(42.0)	
Metastatic sites			**<0.001**
<2	486(75.7)	156(24.3)	
≥2	29(44.6)	36(55.4)	

### Predictive prognosis analysis for variable subgroups in patients with SCLC

Subgroup analysis were performed to assess parameters affecting the prognosis of patients in different groups and identify which group of patients can benefit more from the treatment of chemotherapy cycles≥4 and thoracic radiotherapy. Subgroup analysis was performed in variable KPS, LDH, gender, tumor stage and metastatic sites groups based on multivariate analysis results. The parameters were the same with Table 2. Tumor stage, thoracic irradiation, metastatic sites and LDH were independent prognostic factors of KPS>80 points patients (*p*<0.05 on uni- and multivariate analysis). Tumor stage and chemotherapy cycles were independent prognostic factors in patients with KPS≤80 points. Details of independent prognostic factors in other subgroups analysis can be seen in Table 5.

**Table 5:** Suggested prognostic factors in variable subgroups of SCLC patients

Variables	KPS (points)		LDH (U/L)		tumor stage		metastatic sites		Gender	
	>80	≤80	≤216.5	>216.5	LD	ED	<2	≥2	male	female
Surgery	+/−	−/Ø	+/−	−/Ø	−/Ø	−/Ø	+/−	Ø/Ø	−/Ø	+/−
Stage	+/+	+/+	+/+	+/+	Ø/Ø	Ø/Ø	+/+	Ø/Ø	+/+	+/+
Chemotherapy cycles	+/−	+/+	+/+	−/Ø	−/Ø	+/+	+/+	−/Ø	+/+	+/−
Thoracic irradiation	+/+	+/−	+/+	+/−	+/+	+/−	+/+	−/Ø	+/+	+/−
Metastatic sites	+/+	−/Ø	+/−	+/−	Ø/Ø	+/+	Ø/Ø	Ø/Ø	+/+	+/−
Smoking history	+/−	−/Ø	−/Ø	+/+	+/+	−/Ø	+/−	−/Ø	−/Ø	+/−
NLR	+/−	−/Ø	+/−	−/Ø	−/Ø	+/−	+/−	−/Ø	+/−	−/Ø
LMR	−/Ø	−/Ø	−/Ø	−/Ø	−/Ø	+/−	−/Ø	−/Ø	+/−	−/Ø
Gender	+/−	−/Ø	−/Ø	−/Ø	−/Ø	−/Ø	+/−	−/Ø	−/Ø	−/Ø
Anemia	+/−	−/Ø	−/Ø	−/Ø	−/Ø	−/Ø	+/−	−/Ø	−/Ø	−/Ø
RBC	−/Ø	−/Ø	−/Ø	−/Ø	−/Ø	−/Ø	−/Ø	−/Ø	−/Ø	+/−
LDH	+/+	+/−	Ø/Ø	Ø/Ø	+/+	+/+	+/+	+/+	+/+	+/−
ALP	+/−	−/Ø	−/Ø	−/Ø	−/Ø	−/Ø	+/−	−/Ø	−/Ø	−/Ø
Age	−/Ø	−/Ø	+/−	−/Ø	+/−	−/Ø	+/−	−/Ø	−/Ø	−/Ø
PS	Ø/Ø	Ø/Ø	+/+	+/+	+/+	+/+	+/+	−/Ø	+/+	+/+
WBC	−/Ø	−/Ø	−/Ø	+/+	−/Ø	−/Ø	−/Ø	+/+	−/Ø	−/Ø
Family history	−/Ø	−/Ø	−/Ø	−/Ø	+/+	−/Ø	−/Ø	−/Ø	−/Ø	−/Ø
Lymphocytes	−/Ø	−/Ø	−/Ø	+/−	−/Ø	−/Ø	−/Ø	−/Ø	−/Ø	−/Ø
Neutrophils	−/Ø	−/Ø	−/Ø	+/+	−/Ø	−/Ø	−/Ø	−/Ø	−/Ø	−/Ø

+/+=significant on both univariate and multivariate analysis; +/−=significant on univariate analysis, not significant on multivariate analysis; −/Ø=not significant on univariate analysis and not assessed on multivariate analysis; Ø/Ø= not studied, the parameters used were the same with Table 2 and factors with no statistical significance in variable subgroups were not shown.

**Figure 2:**
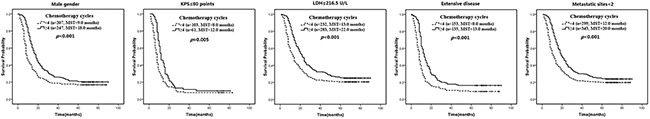
Patients with male gender, KPS≤80 points, LDH≤216 5U/L, extensive disease and metastatic sites<2 can benefit more from the management of etoposide and platinum-based chemotherapy (p<0.05 on uni- and multivariate analysis). MST, median survival time.

**Figure 3:**
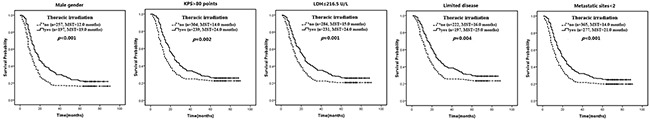
Patients with male gender, KPS>80 points, LDH≤216 5U/L, limited disease and metastatic sites<2 can benefit more from the treatment of thoracic irradiation (p<0.05 on uni- and multivariate analysis). MST, median survival time.

Subgroup analysis showed that patients with male gender, KPS≤80 points, LDH≤216.5U/L, extensive disease and metastatic sites<2 can benefit more from ≥4 chemotherapy cycles (*p*<0.05 on uni- and multivariate analysis, Figure 2). In addition, patients with male gender, KPS>80 points, LDH≤216.5U/L, limited disease and metastatic sites<2 can benefit more from thoracic irradiation (Figure 3). Patients with PS>80 points and female gender can get survival benefits from more cycles of chemotherapy on univariate analysis (*p*<0.05), but it holds no significance on multivariate analysis. Patients with LDH>216.5U/L, limited disease and metastatic sites≥2 can get little benefits from more cycles of chemotherapy (*p*>0.05). Patients with KPS≤80 points, LDH>216.5 U/L, female gender and extensive disease can benefit from thoracic irradiation on univariate analysis (*p*<0.05), but it holds no significant difference on multivariate analysis. Patients with metastatic sites≥2 can get little benefit from the treatment of chemotherapy cycles≥4 and thoracic irradiation (*p*>0.05).

## DISCUSSION

In our study, we find that female patients, KPS>80 points, LDH≤216.5U/L, chemotherapy cycles≥4, thoracic irradiation, metastatic sites**<**2 and limited disease are independent positive prognostic factors for SCLC treated with etoposide and platinum-based chemotherapy. Selected patients can benefit more from the treatment of chemotherapy cycles≥4 and thoracic radiotherapy. Patients with male gender, KPS≤80 points, LDH≤216.5U/L, extensive disease and metastatic sites<2 can benefit more from ≥4cycles of chemotherapy. And patients with male gender, KPS>80 points, LDH≤216.5U/L, limited disease and metastatic sites<2 can benefit more from thoracic radiotherapy. Inflammatory markers of NLR and LMR are associated with the prognosis of SCLC patients, but it holds no statistical significance on multivariate and subgroup analysis. Serum LDH, which can be easily available, still has an independent prognostic values in the condition of more parameters and these results are valuable in clinics.

SCLC is a fatal disease characterized as early recurrence and rapid progression. Many patients progressed quickly and died soon even they received the same treatment strategies. So the identification of patients who can benefit more from the same treatment strategy is important. LDH<216.5 U/L and metastatic sites<2 reflect a low tumor burdens. Subgroup analysis shows chemotherapy cycles≥4 has more significant role in patients with tolerated performance status, male gender, low tumor burdens and relatively extensive disease. Thoracic radiotherapy is more effective in the treatment of patients with better tolerance, limited features, male gender and low tumor burdens. Patients with LDH<216.5 U/L, male gender and metastatic sites<2 can significantly benefit from ≥cycles of chemotherapy and thoracic irradiation. The overall survival benefits of patients with metastatic sites≥2 got from these two treatment is limited, and the treatment in these cases should be carefully considered. Female gender can benefit from these two treatment strategies, but no statistical significance is found on multivariate analysis. Maybe the sample size and grouping of bias can partly account for this result, and the roles of these treatment in female gender still need to be verified. These results can help us identify which treatment strategy is more suitable for variable groups of patients. In addition, it can avoid some additional toxic reactions for patients who can benefit little from ≥4cycles of chemotherapy and thoracic irradiation. This is important for a long-term survival and better quality of life.

Inflammation plays an important role in cancer development, and inflammatory markers of NLR, PLR, LMR et al have been demonstrated to be associated with the prognosis of various malignancies [10, 11, 15–21]. White blood cell (WBC) counts can reflect body inflammatory status, and it plays limited role in the prognosis of the whole SCLC patients (*p*>0.05). However, subgroup analysis shows WBC counts>10×10^9^/L is a positive independent prognostic factor for patients with LDH>216.5 U/L and metastatic sites≥2. WBC may have an important role in patients with high tumor burdens and advanced stage. Similar phenomenon can also be found in previous study and the mechanism remains unknown [1]. High NLR and low LMR are associated with the prognosis of SCLC patients with the presence of more parameters, but no statistical significance is found on multivariate analysis. High NLR reflects a status of neutrophilia or lymphocytopenia. Vascular endothelial growth factor (VEGF), as a proangiogenic factor, is mainly secreted by neutrophils and it plays a critical role in cancer progression [22]. Lymphocytopenia indicates a poor body lymphocyte mediated immune status to cancer. Recent study reveals high NLR is correlated with a distinct cytokine profile related to key biological processes involved in carcinogenesis including an increased expression of interleukin 6 (IL-6), IL-8 and VEGF et al [23]. High NLR and low LMR can promote tumor angiogenesis and inhibit body immunity, leading to a poor prognosis. The potential roles of these inflammatory markers still need to be verified in further research.

LDH has been identified as prognostic factor of SCLC and various malignancies in previous studies [1,5–8,16,21,24–29]. However, the mechanism remains poorly understood. In our study, we found, taking into account of more parameters, LDH still holds statistical significance on a series of analysis. High LDH level is a negative independent prognostic factors of SCLC patients. LDH plays an important role in energy production in various cell types, and an elevated LDH level may promote tumor progression by regulating tumor metabolism [29]. High LDH also reflects a higher tumor burden, the extent of disease and rapid turnover of tumor cells [5,29]. In our study, high LDH is also found to be associated with high ALP level, and patients with elevated ALP show worse treatment tolerance and response in the analysis of overall survival and progression free survival (PFS) [26,29]. In addition, elevated ALP is also correlated with a high tumor burden and can promote tumor progression [29]. Patients with LDH≤216.5 U/L can significantly benefit from the treatment of ≥4 cycles of chemotherapy and thoracic irradiation on subgroup analysis. These results may have some hints for our clinical decisions for the treatment of patients with variable LDH levels.

There are some limitations of our study. First, it is a single constitutional retrospective analysis and we exclude many patients treated with best supportive care or other treatment strategies in order to assess the prognostic roles of parameters in patients treated with etoposide and platinum-based chemotherapy. That is why there are more limited disease patients in our cohort. Second, we do not assess the impact of these factors on PFS for the absence of PFS information in some patients. In addition, patients number in some groups is relatively small. We will pay more attention to these details in our further study.

In conclusion, female patients, KPS>80 points, LDH≤216.5U/L, chemotherapy cycles≥4, thoracic irradiation, metastatic sites**<**2 and limited disease are independent positive prognostic factors for patients treated with etoposide and platinum-based chemotherapy. Patients with male gender, KPS≤80 points, LDH≤216.5U/L, extensive disease and metastatic sites<2 can benefit more from ≥4cycles of chemotherapy. And patients with male gender, KPS>80 points, LDH≤216.5U/L, limited disease and metastatic sites<2 can benefit more from thoracic radiotherapy. Further retrospective and prospective studies are urgently needed to provide evidence-based recommendations for our clinics.

## MATERIALS AND METHODS

### Patients

We conducted a retrospective analysis on 1126 cases with pathologically confirmed SCLC who were registered and followed up in our hospital between January 2008 and January 2010. Only patients treated with etoposide and platinum-based chemotherapy were included in this study. The criteria for entry into our study were as follows: 1. histologically or cytologically proved SCLC; 2. no prior anti-tumor therapies; 3. age≤75 years; 4. complete follow-up information; 5. combined routine blood test and liver function examinations. Finally, 707 patients were included in our study. Our study was approved by the Ethics Committee of Harbin Medical University Cancer Hospital and written informed consent was obtained from each patient. Limited disease (LD) is defined as disease confined to the ipsilateral chest within a single radiation field, while extensive disease (ED) was defined as disease beyond the ipsilateral hemithorax including malignant pleural, pericardial effusion, or hematogenous metastasis.

### Data collection

The following parameters were collected and evaluated for prognostic impact: age (≤65 or >65 years), gender, KPS (Karnofsky Performance Status), smoking history, family history of cancer, serum red blood cell (RBC), platelet (≤300,000 or >300,000/μl), white blood cell (WBC, ≤10 or >10×10^9^/L), hemoglobin, neutrophil (≤4.95 or >4.95×10^9^/L), lymphocyte (≤1.65 or >1.65×10^9^/L), monocyte (≤0.495 or >0.495×10^9^/L), NLR (≤3.18 or >3.18), PLR (≤176.5 or >176.5), LMR (≤2.615 or >2.615), aspartate aminotransferase (AST, ≤40 or >40 U/L), alanine aminotransferase (ALT, ≤40 or >40 U/L), LDH (≤216.5 or >216.5 U/L), ALP (≤119.5 or >119.5 U/L), albumin (≤40 or >40 g/L), globulin (≤35 or >35 g/L), surgery, chemotherapy cycles, thoracic irradiation, metastatic sites and tumor stage at initial diagnosis. A low serum RBC level was defined as a red blood cell count <3.5 or 4.0×10^9^/L in females and males, respectively. Anemia is defined as a hemoglobin value<12.0 g/dl in men and 11.0 g/dl in women. The NLR, PLR, and LMR values were calculated using the neutrophil, platelet, lymphocyte and monocyte counts as the ratio of neutrophil and platelet counts to lymphocyte or lymphocyte counts to monocyte, respectively.

### Statistical analysis

Mean values±standard deviation (SD) or median and range were calculated for continuous variables. The optimal cut-off point for neutrophil, lymphocyte, monocyte, NLR, PLR, LMR, LDH and ALP were calculated by a receiver operating characteristic (ROC) curve analysis. Survival outcomes were dichotomized by survival status (alive or dead) until a defined time of the median survival time of 15.0 months in the ROC curve analysis. The overall survival time was defined from diagnosis to death or the last follow-up of April 2015 if the patients were still alive. The survival curves and 95% confidence intervals (CIs) were obtained using the Kaplan-Meier method, and the survival was compared by log-rank test. The relationship between LDH and the clinicopathological features were evaluated using the chi-squared test. Univariate analysis was used to examine the potential prognostic roles of all factors. The prognostic factors with *p-*value≤0.05 in the univariate analysis were examined in the multivariate analysis. SPSS 16.0 statistical software was used for statistical analysis. All *P*-values≤0.05 were considered statistically significant.
